# Identification of drought-responsive microRNAs in *Medicago truncatula *by genome-wide high-throughput sequencing

**DOI:** 10.1186/1471-2164-12-367

**Published:** 2011-07-15

**Authors:** Tianzuo Wang, Lei Chen, Mingui Zhao, Qiuying Tian, Wen-Hao Zhang

**Affiliations:** 1 State Key Laboratory of Vegetation and Environmental Change, Institute of Botany, the Chinese Academy of Sciences, Beijing 100093, PR China; 2 Graduate University of the Chinese Academy of Sciences, Beijing 100049, PR China

## Abstract

**Background:**

MicroRNAs (miRNAs) are small, endogenous RNAs that play important regulatory roles in development and stress response in plants by negatively affecting gene expression post-transcriptionally. Identification of miRNAs at the global genome-level by high-throughout sequencing is essential to functionally characterize miRNAs in plants. Drought is one of the common environmental stresses limiting plant growth and development. To understand the role of miRNAs in response of plants to drought stress, drought-responsive miRNAs were identified by high-throughput sequencing in a legume model plant, *Medicago truncatula*.

**Results:**

Two hundreds eighty three and 293 known miRNAs were identified from the control and drought stress libraries, respectively. In addition, 238 potential candidate miRNAs were identified, and among them 14 new miRNAs and 15 new members of known miRNA families whose complementary miRNA*s were also detected. Both high-throughput sequencing and RT-qPCR confirmed that 22 members of 4 miRNA families were up-regulated and 10 members of 6 miRNA families were down-regulated in response to drought stress. Among the 29 new miRNAs/new members of known miRNA families, 8 miRNAs were responsive to drought stress with both 4 miRNAs being up- and down-regulated, respectively. The known and predicted targets of the drought-responsive miRNAs were found to be involved in diverse cellular processes in plants, including development, transcription, protein degradation, detoxification, nutrient status and cross adaptation.

**Conclusions:**

We identified 32 known members of 10 miRNA families and 8 new miRNAs/new members of known miRNA families that were responsive to drought stress by high-throughput sequencing of small RNAs from *M. truncatula*. These findings are of importance for our understanding of the roles played by miRNAs in response of plants to abiotic stress in general and drought stress in particular.

## Background

Endogenous small interfering RNAs (siRNAs) and microRNAs (miRNAs) are the two most abundant classes of plant small RNAs (sRNAs). The small RNAs are processed in the nucleus from longer precursor transcripts that form distinct secondary structures. The miRNAs down-regulate gene expression by targeting specific messenger RNAs (mRNAs) in both plants and animals [1,2].

miRNAs were initially discovered in *Caenorhabditis elegans *as developmental timing regulators in 1994 [3]. The existence of miRNAs in organisms including plants, mammals and virus has widely been recognized. The biogenesis of miRNAs in plants is a multi-step enzymatic process involving incorporation of miRNAs into the RNA-induced silencing complex (RISC), and then miRNAs bind target mRNAs to direct for cleavage miRNAs with near perfect complementarity and/or inhibiting translation of those with lower complementarity [4-7]. There has been ample evidence demonstrating that miRNAs play a regulatory role in diverse biochemical and physiological processes in plants [1,4]. For instance, miRNAs have been shown to play a role in the modulation of the processes associated with growth and development in plants, including leaf morphogenesis, floral organ and root development [6,8-13]. In addition, recent studies also revealed that miRNAs are involved in responses of plants to various abiotic and biotic stresses. These include drought [14-18], cold [19-21], salinity [22], nutrient starvation [23-26], oxidative stress [27], submergence [28], UV-B radiation [29,30] and virus [31,32].

Plants are hardly grown under optimal conditions, and have to frequently suffer from drought stress due to shortage of water supply. Not unexpectedly, the involvement of miRNAs in response of plants to drought has been evaluated in several plant species. A number of miRNAs have been identified to be associated with response to drought stress in several species such as *Arabidopsis thaliana *[14,33] and *Oryza sativa *[17,18]. For instance, Zhou *et al*. (2010) identified 11 down-regulated miRNAs and 8 up-regulated miRNAs in response of rice to drought [18]. It has been reported that miR169g is the only member induced by drought in the miR169 family of rice and its expression is regulated by drought [17]. Li *et al*. (2008) found that miR169a and miR169c are substantially down-regulated by drought, leading to the enhanced resistance to drought in Arabidopsis because one of the miR169's targets, NFYA5 (Nuclear Factor YA5), is a crucial transcription factor regulating the expression of a number of drought stress-responsive genes [14]. Recent studies also demonstrated that miRNAs play a role in control of drought resistance in maize by modulating the expression of MAPK (mitogen-activated protein kinase), PLD (phospholipase D), PDH (praline dehydrogenase) and POD (peroxidase) [16]. More recently, Trinadade *et al*. (2010) reported that miR398 and miR408 are up-regulated by water deficit in *Medicago truncatula*, leading to a down-regulation of their target genes of *COX5b*, *CSD1 *and plantacyanin [15]. In *Triticum dicoccoides*, 13 miRNAs are differentially regulated in response to short-term drought stress by miRNA microarray chip [34]. Despite of numerous studies on the involvement of miRNAs in abiotic stress in general and drought stress in particular, there has been no report on the systemic identification of drought-responsive miRNAs in leguminous plants at the global genome-level by high-throughout sequencing.

Leguminous plants account for one third of primary crop production in the world and are important sources for the consumption of human and animals [35]. Drought stress is one of the most frequently occurred environmental stresses that limit crop yield world-wide. *Medicago truncatula *is an annual legume species distinguished by its small diploid genome and easy transformation, and is a model plant to study functional genomics of legume plants [36]. Although numerous miRNAs have been identified in various plant species and the mechanisms underlying their actions are being unravelled, the discovery of new miRNAs in plants on a genome-wide scale is essential for functional characterization of miRNAs. The traditional sequencing of relatively small-size cDNA libraries of plant sRNAs from Arabidopsis, rice and poplar with Sanger method has led to the conclusions that plant miRNAs are highly conserved [37]. However, a small number of miRNAs was not detected in the genomes of some species, indicating that miRNAs may evolve more recently [38]. In addition, the non-conserved miRNAs are often expressed at a lower level than the conserved miRNAs, thus many non-conserved miRNAs cannot be detected in small-scale sequencing studies. In this context, high-throughput sequencing has been used to identify non-conserved miRNAs in several species [20,39-53]. To understand the role of miRNAs in response of plants to drought stress, we identified a number of conserved and non-conserved miRNAs that were responsive to drought by high-throughput sequencing, and their potential role in drought tolerance was discussed.

## Results

### High-throughput sequencing of small RNAs from M. truncatula

**Figure 1:**
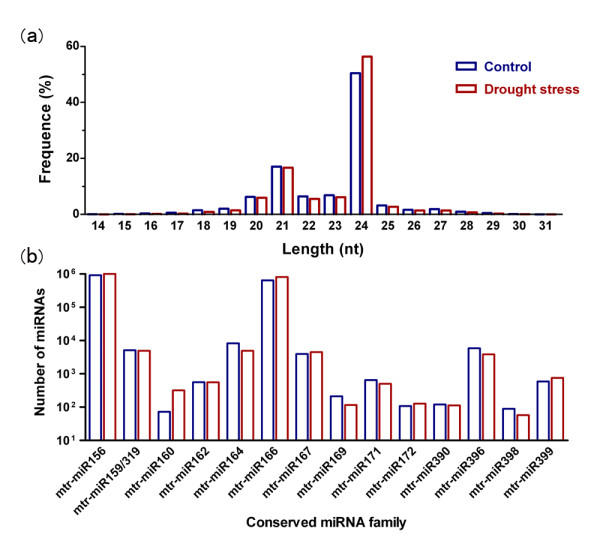
**Distribution of small RNAs from control and drought stress libraries**. The size distribution of small RNAs is shown in panel (a) with frequency. The absolute miRNA number in different conserved families is shown in panel (b).

Two sRNA libraries in which *M. truncatula *seedlings were treated with drought stress (DS) and without stress, control (CK) constructed from shoots of seedlings grown under the two treatment-regimes were sequenced by an Illumina-Solexa sequencer. High-throughput sequencing of CK and DS libraries led to generation of 6,808,877 and 6,874,742 primary reads, respectively. There were 6,420,234 clean reads (2,685,754 unique sequences) for CK and 6,505,965 clean reads (2,937,417 unique sequences) for DS after initial processing (Table 1). The length distribution of reads showed that the majority of the reads was 20-25 nt in size, of which the class of 24 nt was the most abundant group followed by the group of 21 nt class (Figure 1a).

**Table 1:** Statistics of sRNA sequences for control (CK) and drought stress (DS) libraries

	Number of reads	Number of unique sequences
Control (CK)		
Primary reads	6,808,877	
Clean reads	6,420,234	2,685,754
Sequences mapped to the genome	4,875,034	1,713,480
match known miRNAs	509,981	283

Drought stress (DS)		
Primary reads	6,874,742	
Clean reads	6,505,965	2,937,417
Sequences mapped to the genome	4,819,359	1,867,375
Match known miRNAs	588,990	293

The common/specific sequences were analyzed between CK and DS libraries for the total and unique sequences. There were 19.14% and 44.95% specific sequences in the DS library for total and unique sequences, respectively.

After initial processing, the high-quality small RNA reads were mapped to the *M. truncatula *genome sequence (Mt3.0) using SOAP [54]. The number of total and unique sequences that matched genome was 4,875,034/1,713,480 and 4,819,359/1,867,375 in the two libraries, respectively (Table 1).

### Identification of known miRNAs

The known miRNAs in *M. truncatula *were identified by comparing with the up-dated miRNAs database (miRBase 17, released in April 26, 2011) [55]. We identified 283 and 293 known miRNAs in the two libraries, respectively (Table 1). Sixty-six members belonging to 14 conserved miRNA families were obtained after removing those miRNAs whose expression levels are too low to be analysed for differential expression. Of these families, the most abundant two reads were miR156 and miR166. Among the identified miRNAs, miR399 contained the most members, including miR399a-i and miR399k-q (Figure 1b).

In addition to the conserved miRNAs, we also identified 137 non-conserved miRNAs that belong to 44 miRNA families (Additional file 1) after removing miRNAs with expression levels that were too low to be analysed for differential expression. Among the 137non-conserved miRNAs, some were legume-specific. The most abundant miRNA was miR2086 followed by miR2111 in the control (CK) library. In the drought stress (DS) library, miR2111 was the most abundant, followed by miR1507. In addition, miR2630 was found to have the most members in the two libraries (miR2630a-y).

### Identification of new miRNAs/new members of known miRNA families

**Figure 2:**
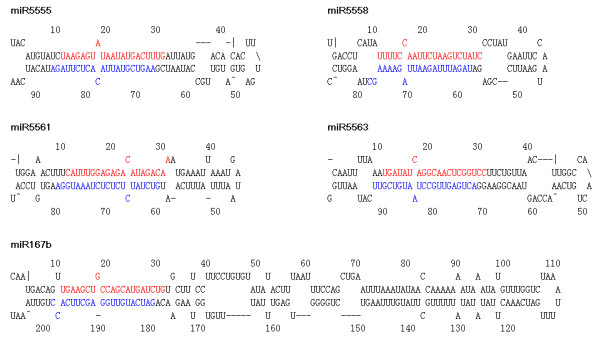
**Representatives of predicted precursors' hairpin structures of new miRNA/new members of known miRNA families**. The mature miRNA and miRNA* sequences are coloured in red and blue, respectively. All the predicted hairpin structures of these miRNA precursors are shown in Additional file 3.

The formation of stable hairpin structure is one of the essential features for identification of new miRNAs [56]. To identify new miRNAs and new members of known miRNA families, we pooled the reads obtained from the two libraries, and identified the miRNAs by folding the sequences of potential miRNA precursors using the mfold web server [57]. We obtained 238 potential miRNA candidates (Table 2, Additional file 2). In addition to the hairpin structure, detection of miRNA*s is another proof that has been widely used to confirm the new miRNAs [58]. Among the 238 potential miRNA candidates, we found 29 miRNAs with complementary miRNA*s (Table 2, Figure 2, Additional file 3), indicating that these candidate miRNAs are likely to be new miRNAs or new members of known miRNA families in *M. truncatula*. The counts of several miRNA*s of these miRNAs were low (Table 2). This may make the annotation of these new miRNAs questionable. However, no requirement on the counts of several miRNA*s was given in the most recent criteria for annotation of plant miRNAs [58]. Therefore, these miRNAs were still considered to be new miRNAs or new members of known miRNAs. A similar identification of new miRNAs has also been reported by others in the literature. For instance, candidate miRNAs with only one miRNA* have been identified as new miRNAs in rice [59].

**Table 2:** New miRNAs and new members of known miRNA families with miRNA*s in *M. truncatula*

miRNAs	Sequence (5'-3')	Length (nt)	Counts of miRNAs/miRNA*s	Location	Arm	Length of precursors (nt)	MFE (kcal/mol)
miR5213	uacgugugucuucaccucugaa	22	15045/44	MtChr2:16737666:16737796:+	5'	131	-43.1
miR5274b	auaugacggaguguaaaugcc	21	29/2	MtChr4:11424365:11424459:-	5'	95	-66.3
miR5554a	ugugcaucuugaacaaugguau	22	134/3	MtChr1:31285750:31285854:-	5'	105	-52.8
miR5554b	ugugcaucuugaacaaugguau	22	134/3	MtChr4:41013393:41013497:-	5'	105	-57.3
miR5554c	ugugcaucuugaacaaugguau	22	134/1	MtChr7:19742691:19742795:-	5'	105	-53.3
miR5555	uaagaguauaauaugacuuug	21	40664	MtChr1:12905862:12905956:-	5'	95	-47.5
miR5556	ugaugacggaagaaauccaaa	21	155/1	MtChr4:23199944:23200064:-	3'	121	-54.8
miR5557	ugcuuccuuaguacuuguuga	21	5/3	MtChr5:6026167:6026481:+	3'	315	-96.7
miR5558	uuuuccaauucuaagucuauc	21	139/8	MtChr5:20521087:20521172:+	5'	86	-33
miR5559	uacuuggugaauuguuggauc	21	2124/2	MtChr6:937306:937443:+	5'	138	-48.8
miR5560	ugccggcucaaugaaugcggag	22	62/2	MtChr6:4747428:4747538:+	3'	111	-53.8
miR5561	cauuuggagagacauagacaa	21	449/3	MtChr7:30170572:30170660:-	5'	89	-42.9
miR5562	uguggagucuuuugcaugaag	21	16/1	MtChr7:32194444:32194667:-	5'	224	-56.4
miR5563	ugauaucaggcaacucggucc	21	12/1	MtChr8:32699524:32699624:-	5'	101	-52.9

miR156j	uugacagaagagggugagcaca	22	10341/23	MtChr1:3228429:3228554:+	5'	126	-44.4
miR167b	ugaagcugccagcaugaucug	21	40937/1	MtChr7:34009590:34009796:+	5'	207	-78.7
miR168c	ucgcuuggugcaggucgggaa	21	56024/1434	MtChr5:10397699:10397824:+	5'	126	-68.8
miR172b	agaaucuugaugaugcugcau	21	35344/63	AC235487:197944:198077:+	3'	134	-55.5
miR172c	agaaucuugaugaugcugcau	21	35386/8	AC233663:10168:10308:+	3'	141	-52.8
miR408	augcacugccucuucccuggc	21	75/55	MtChr1:21952074:21952198:+	3'	125	-45.1
miR2111u	uaaucugcauccugagguuua	21	413/23	MtChr7:14331003:14331117:-	5'	115	-50.2
miR2111v	uaaucugcauccugagguuua	21	1192/41	MtChr7:14356392:14356499:-	5'	108	-48.4
miR2592a	gaaaaacaugaaugucgagcg	21	28/1	MtChr1:27380857:27381050:+	3'	194	-89.4
miR2592bl	uggcaaguuugaauuuaccuca	22	43/1	MtChr4:18239825:18239954:+	5'	130	-69.3
miR2592bm	ggaaaacaugaaugucgggug	21	918/294	MtChr5:3041012:3041204:+	3'	193	-104.4
miR2592bn	ggaaaacaugaaugucgggug	21	530/160	MtChr5:8400182:8400375:+	3'	194	-111.8
miR2619b	auauguuuugauucuuuggca	21	9/3	MtChr4:6335670:6335840:+	5'	171	-94.7
miR2643b	uuugggaucagaaauuagaga	21	361/11	MtChr5:41836449:41836558:-	3'	110	-38.8
miR4414a	agcugcugacucguugguuca	21	663/45	MtChr1:30518803:30518924:+	5'	122	-50.5

Twenty-two out of 29 new miRNAs/new members of known miRNA families had length of 21 nt, while remaining miRNAs had length of 22 nt. The majority of the miRNAs started with a 5' uridine, a hallmark of miRNAs [60]. The minimum free energy (MFE) for hairpin structure of miRNA precursors was lower than -30 kcal/mol (Table 2). This feature is in agreement with other reported values in the literature [61]. The length of new miRNAs' precursors ranged from 86 nt to 315 nt (Table 2). These values are comparable to those reported for *M. truncatula *in the literature [42,51,52,62,63].

### Response of known miRNAs to drought stress

**Figure 3:**
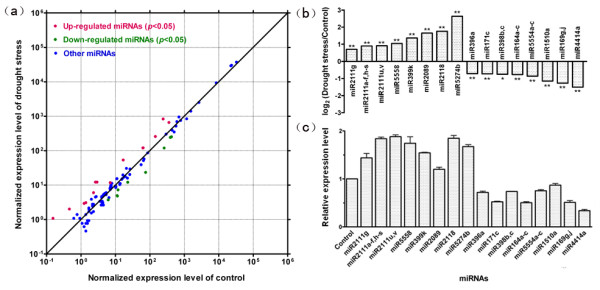
**Differential expression analysis between control and drought stress**. Data points at the upper and lower side of the slope line represent up- and down-regulated miRNAs in panel (a), respectively. The changes in miRNAs for up- and down-regulated ones are greater than 1.5-fold. Other miRNAs include those that are not responsive to drought stress and those changes induced by drought stress at p > 0.05. Expression of control and drought stress was normalized on the basis of 1 M reads. Differential expression of known miRNAs in response to drought stress is shown in panel (b). The positive and negative values mean miRNAs whose expression was stimulated and suppressed by drought stress, respectively. * and ** mean significant difference between control and drought stress at 0.01 < p ≤ 0.05 and p ≤ 0.01, respectively. The relative expression level of miRNAs measured by RT-qPCR in response to drought stress is shown in panel (c).

To identify drought-responsive miRNAs, the normalized expression of miRNAs in the two libraries (control, CK and drought stress, DS) was compared. Based on the results of high-throughput sequencing, we selected those miRNAs with changes in expression levels being greater than 1.5-fold in response to drought treatment (Figure 3a and 3b, Additional file 4) to validate the expression patterns by real-time quantitative PCR. As shown in Figure 3b and 3c, expression patterns of drought-responsive miRNAs from high-throughput sequencing and RT-qPCR exhibited similar results. Results from both the methods showed that 22 members in 4 miRNA families, i.e., miR399, miR2089, miR2111 and miR2118, were up-regulated in response to the drought stress. Conversely, 10 members belonging to 6 miRNA families, i.e., miR164, miR169, miR171, miR396, miR398 and miR1510, were down-regulated in response to drought stress (Figure 3b and 3c). These miRNAs have been reported to be involved in diverse cellular processes in plants [14,17,22,52]. The known target genes of these miRNAs and their function annotations were summarized in Table 3. For those miRNAs whose targets are not known, we predicted their targets using the psRNATarget http://bioinfo3.noble.org/psRNATarget/ and the srna-tools http://srna-tools.cmp.uea.ac.uk/plant/[64], and the results are given in the Table 4. Among these miRNAs, several miRNAs have been reported to be involved in abiotic stresses. For example, miR399 and miR2111 have been reported to be up-regulated by phosphate starvation [23,65], while miR169 with target of CCAAT Binding Factor is down-regulated in response to drought stress [14,17].

**Table 3:** The known targets of drought-responsive miRNAs and their function annotations

miRNA families	Expression pattern	Targets	Functions and responsiveness	References
miR164	down	NAC domain transcription factors	lateral root development	[9]
miR169	down	CCAAT Binding Factor (CBF)	response to drought, cold and salinity, nodule development	[14,15,17,18,22,33,74]
miR171	down	GRAS transcription factors	response to drought, cold and salinity, nodule morphogenesis, floral development	[5,18,33]
miR396	down	Growth Regulating Factor (GRF)	response to drought and salinity, cell proliferation	[18,52,75]
miR398	down	Cu/Zn superoxide dismutases (CSD1, CSD2)	response to oxidative stress	[15,27]
miR399	up	PHO2/ubiquitin conjugating enzyme	balance of phosphorus	[23]
miR2118	up	TIR-NBS-LRR domain protein	response to drought, cold, salinity, and ABA	[51,76]

**Table 4:** Predicted targets of drought-responsive miRNAs

miRNAs	Expression pattern	Predicted targets
miR1510a	down	1. pyruvate decarboxylase (PDC) isozyme 1
		2. concanavalin A-like lectin/glucanase
		3. F-box protein
miR2089	up	NB-ARC domain protein
miR2111a-s	up	1. calcineurin-like phosphoesterase
		2. membrane protein SAK

miR2111u, v	up	1. calcineurin-like phosphoesterase family protein
		2. membrane protein SAK
miR5274b	up	DNA-damage-repair/toleration protein
miR5554a-c	down	polynucleotidyl transferase, Ribonuclease H fold
miR5558	up	1. initiation factor eIF-4 gamma
		2. homeodomain-related POX

### Response of new miRNAs/new members in known miRNA families to drought stress

We further examined whether the 29 new miRNAs/new members of known miRNA families were responsive to drought stress using the same method as the known miRNAs. The miRNAs with changes in expression levels being greater than 1.5-fold and *p-*values less than 0.05 are presented in Table 4. Results from high-throughput sequencing and RT-qPCR showed that, among the 14 new miRNAs, 5 miRNAs were responsive to drought stress with miR5274b and miR5558 being up-regulated, while miR5554a-c being down-regulated. In addition, among the 15 new members of known miRNA families, 3 miRNAs were responsive to drought stress with miR2111u, v being up-regulated and miR4414a being down-regulated (Figure 3b and 3c, Table 4).

## Discussion

### High-throughput sequencing of M. truncatula small RNAs

High-throughput sequencing has been used to study miRNAs at whole genome level in several plant species, including Arabidopsis [39,40], rice [43], wheat [44], soybean [45], cotton [46], grapevine [53], Medicago [42,51,52], Brachypodium [20], tomato [41], populus [47], citrus [48], peanut [49], Porphyra [50]. However, most of the studies on high-throughput sequencing miRNAs have focused on miRNAs under non-stressed, normal growth conditions, and only a few studies have compared miRNAs under control conditions to those under conditions of abiotic stresses by high-throughput sequencing plant miRNAs [20,43,44]. For instance, Sunkar *et al*. (2008) reported a total of 714,202 reads in rice seedlings from three independent libraries (control, drought and salt stress libraries) [43]. Twenty-eight and 12 miRNAs in response to cold stress in Brachypodium [20,44] and heat stress in wheat [44] have been identified by high-throughput sequencing, respectively. In the present study, we constructed two sRNA libraries, non-stressed, control and drought stressed in legume model plant, *M. truncatula *and identified known and new miRNAs that were responsive to drought stress.

The successful application of high-throughput sequencing technology to systemically identify plant miRNAs has greatly advanced our knowledge on the functions of miRNAs in plants in recent years. There have been several reports on the identification of miRNAs in *M. truncatula *in the literature. For instance, 26,656 and 844,110 sRNA reads were reported in two recent studies from *M. truncatula *by Jagadeeswaran *et al*. (2009) and Lelandais-Briere *et al*. (2009), respectively, with 97,028 unique sequences being obtained by the latter studies [51,52]. However, in both cases, sRNAs were sequenced using Roche 454 sequencer, and the total reads and unique sequences obtained from these studies are much less than we reported in the present study (Table 1). In a study by Szittya *et al*. (2008), the authors obtained 3,948,871 reads and 1,563,959 unique sequences from two sRNAs libraries of *M. truncatula *using the same high-throughput sequencing technology (Illumina-Solexa) [42]. In contrast to these reported findings, we got a total of 13,683,619 reads and 4,878,445 unique sequences from two libraries of *M. truncatula*. Therefore, our results contain more reads than those previously reported for miRNAs in *M. truncatula *in the literature. Moreover, the high-throughput sequencing technology used in the present study allows us to identify the miRNAs with low abundance, thus accounting for the greater unique sequences found in our studies. In addition to the greater amounts of miRNAs sequencing data, the use of most updated miRBase also contributes to our identification of more known miRNAs in *M. truncatula*. The available database for *M. truncatula *contains more miRNAs than those for other plant species. For example, in the database, there are 19 miRNAs and 41 miRNAs for Brachypodium and wheat, respectively, while there are 375 miRNAs in the database for *M. truncatula *(miRBase 17). In addition, to the best of our knowledge, we are the first one to use the *M. truncatula *genome sequence Mt3.0 to analyze miRNAs in this species. The more genomic information in Mt3.0 than Mt2.0 may also contribute to predicting more new miRNAs in *M. truncatula*.

### Drought-responsive miRNAs

Plant miRNAs have a strong propensity to target genes associated with development, particularly those genes encoding transcription factors and F-box proteins [25]. In the present study, we found that the known or predicted targets of miR164, miR169, miR171, miR396, miR1510 and miR5558 were either transcription factors or F-box proteins (Table 3, 4). Under drought stress, an increase in root/shoot ratio was found in *M. truncatula *(data not shown). miR164 has been reported to regulate root development by a homeostatic mechanism to clear NAC1 mRNA, leading to down-regulation of auxin signals. It has been shown that decreases in NAC1 mRNA levels due to inducible expression of miR164, thus resulting in reduction in lateral root emergence in Arabidopsis [9]. Therefore, it is conceivable that the suppression of miR164 expression may contribute to the increase in root/shoot ratio under drought stress.

Several studies have revealed that miR169 is responsive to abiotic stresses such as drought, cold and salinity in different species [14,15,17,22,33]. Under drought stress, miR169 exhibited different expression patterns among different species. For example, in Arabidopsis, miR169 is down-regulated by drought stress through an ABA-dependent pathway, resulting in accumulation of NFYA5 with high affinity and sequence specificity for the CCAAT box, which is crucial for the expression of a number of drought-responsive genes [14]. In contrast to Arabidopsis, miR169g in rice is up-regulated by drought [17,18]. In rice, DREs (dehydration-responsive element) as the upstream of *MIR169g *are regulated by cold and drought stress, thus leading to up-regulation of miR169g [17]. Trindade *et al*. (2010) reported that the expression of miR169 in leaves of *M. truncatula *is not responsive to drought stress [15]. In contrast, our results from both the high-throughput sequencing and RT-qPCR showed that miR169 was down-regulated under drought stress in *M. truncatula *(Figure 3b and 3c, Additional file 4). This discrepancy may result from the difference in degree of drought stress exposed to plant materials in the two studies. For instance, in the present study, the drought stressed samples were collected after exposing of plants to drought for varying period (6, 8, 10 and 12 d after withholding water), thus our samples included plant materials suffering from a wide range of drought stress. This type of stress has been used in study of molecular response of plants to drought stress [66,67]. In the studies of Trindade *et al*. (2010), water stress was exposed by suppressing water supply with relative water content in leaves being approx. 50% and 30%, respectively [15]. In the present paper, the drought stressed leaves with relative water content of 87.4%, 78.8%, 75.2% and 68.3% were used for analysis of miRNAs. Therefore, our samples were mainly those suffering from mild and moderate water stress compared to those of Trindale *et al*. (2010). If miR169 is responsive to mild and/or early drought stress exclusively, the responsiveness of miR169 may not be detected by the more severe drought stress used by the authors [15]. In addition to withholding water supply, several studies on effect of drought on miRNAs also treated plants with PEG or mannitol for varying period [17,33]. It is difficult to compare the natural drought stress with PEG-induced and/or mannitol osmotic stress as the two treatments may differ in induction of water stress in terms of rapidity and severity.

Accumulation of reactive oxygen species (ROS) is a common phenomenon in response of plants to abiotic stress. The accumulated ROS damage nucleic acid, oxidize proteins and cause lipid peroxidation [68,69]. Superoxide dismutases (SODs) detoxify superoxide radicals. The targets of miR398 are two Cu/Zn superoxide dismutases (cytosolic CSD1 and chloroplastic CSD2), and miR398 expression was reported to be down-regulated transcriptionally by oxidative stresses [27]. Oxidative stress often occurs concurrently with drought stress. In the present study, we found that miR398 was down-regulated under drought stress. This would lead to increases in activities of SODs. The drought-induced down-regulation of miR398 in *M. truncatula *is consistent with the results in maize [16], but it is contrast to the results reported by Trindale *et al*. (2010) [15] and Kantar *et al*. (2011) [34]. The differences in the expression of miR398 among different studies may results from differences in species, extent and duration of drought stress in different studies.

Plants suffering from water-deficit often display reduced uptake of mineral nutrients. In this context, miR399 negatively regulates the concentration of inorganic phosphate (Pi) by targeting PHO2, a type of E2 conjugase, and overexpression of miR399 in Pi-replete conditions represses expression of E2 conjugase, leading to an increase in Pi concentration in leaves in Arabidopsis [23]. Expression of miR399 is reduced under Pi-deprived conditions to facilitate accumulation of Pi in plants. It has been verified that miR2111 is up-regulated by Pi starvation [65]. In our study, the expression miR399 and miR2111 was similar under drought stress. These results indicate that the function of miR2111 may be as important as miR399 in regulation of nutrient acquisition.

Exposure of plants to a moderate stress induces resistance to other stresses, a phenomenon known as cross adaptation, which has been found in different combinations of stresses [70]. For example, osa-miR821 isolated from virus-infected rice tissues is also expressed in roots of salt-stressed plants, while it is not expressed in healthy, non-stressed plants [71]. Under drought stress, we found that there was up-regulation of miR2089 and miR2118, whose targets may be proteins associated with disease resistance. It is envisaged that these miRNAs may enhance the ability of drought tolerance through unknown mechanisms associated with cross adaptation in plants. Future work aiming at functional elucidation of these miRNAs is warranted by over-expressing these miRNAs in *M. truncatula*.

## Conclusions

We obtained a total of 13,683,619 reads from two small RNA libraries of *M. truncatula *by high-throughput sequencing. Twenty-two members in 4 miRNA families and 10 members belonging to 6 miRNA families were found to be up-regulated and down-regulated in response to drought stress by both high-throughput sequencing and RT-qPCR. In addition, we also predicted 29 new miRNAs/new members of known miRNA families, of which 8 miRNAs were responsive to drought stress by high-throughput sequencing and RT-qPCR. These findings provide valuable information for further functional characterization of miRNAs in response to abiotic stress in general and drought stress in particular in legume plants.

## Methods

### Plant materials and drought stress treatment

Four *Medicago truncatula *(cv Jemalong A17) seedlings were grown in a pot (diameter 10 cm) filled with vermiculite: peat soil (2:1) under controlled conditions (26°C day/20°C night, 14-h photoperiod, and 50% relative humidity). Drought stress was initiated by withholding water supply to 3-week-old seedlings for varying periods after seedlings were fully watered. The severely wilted leaves appeared on the 14th day after the water withholding. The soil water content was reduced from approx. 60% to 8.1% during the drought stress. Shoots under drought stress were harvested after withholding of water for 6, 8, 10 and 12 d, and mixed averagely as drought treatment materials (DS). The relative water content for leaves collected after 6, 8, 10 and 12 d drought stress was 87.4%, 78.8%, 75.2% and 68.3%, respectively. At the same time, shoots of *M. truncatula *seedlings grown under normal watering conditions were also harvested as control (CK). The relative water content for control leaves was 91.4%. The measure method of relative water content was described by Catsky (1960) [72].

### Small RNA libraries construction for high-throughput sequencing

To construct small RNA libraries, total RNA was extracted from shoots of control (CK) and drought stress (DS) using the Trizol (Invitrogen) according to the manufacturer's protocols. For each sample, the 18-30 nt small RNAs were ligated with 5' and 3' RNA adapter by T4 RNA ligase (TaKaRa) after they were purified by electrophoretic separation on a 15% TBE-urea denaturing PAGE gel, and at each step purified by urea PAGE gel electrophoretic separation. The RNA was subsequently transcribed to single-stranded cDNA using Superscript II reverse transcriptase (Invitrogen). Thereafter the cDNA was used as templates for double-stranded cDNA synthesis by PCR amplification using primers that anneal to adapters. The purified DNA was sequenced on a Solexa sequencer (Illumina). The raw data have been submitted to Gene Expression Omnibus (GEO, http://www.ncbi.nlm.nih.gov/projects/geo/) and the accession number is GSE29154.

### Bioinformatics analysis

After the sequence tags from Solexa sequencing went through the data cleaning by removing the low quality tags (i.e., tags less than 18 nt and tags whose adaptors were null) and contaminants (adaptors and polyA), the length distribution and common/specific sequences between two samples were analyzed. After removing adaptor sequences of the left high-quality small RNA reads with exact matches to the adaptor sequences, reads were mapped to the *M. truncatula *genome sequence (Mt3.0) downloaded from the website http://www.medicagohapmap.org/downloads_genome/Mt3 using SOAP [54]. rRNAs, tRNAs, snRNAs and snoRNAs were removed from the matched sequences through BLASTn search [73] using NCBI Genebank database http://www.ncbi.nlm.nih.gov/blast/Blast.cgi/ and Rfam database http://www.sanger.ac.uk/Software/Rfam/. Mismatches were not allowed in the above two approaches. The unique sequences left were aligned with known miRNAs from miRBase 17 http://www.mirbase.org/[55]. The potential candidate miRNAs were identified by folding the flanking genome sequence of unique small RNAs using the mfold web server [57]. Parameters were set based on the criteria for annotation plant miRNAs [58]. Target predictions were performed using the psRNATarget http://bioinfo3.noble.org/psRNATarget/ and the srna-tools http://srna-tools.cmp.uea.ac.uk/plant/[64], through aligned with genome of *M. truncatula *and *A. thaliana*.

### Differential expression analysis of miRNAs under the drought stress

The frequency of miRNAs of two libraries was normalized to one million by total number of miRNAs in each sample (Normalized expression = Actual miRNA count/Total count of clean reads*1,000,000). miRNAs whose normalized expression of two libraries is smaller than one were removed, because their expression levels are too low. The fold change between treatment and control was calculated as: Fold-change = log_2 _(DS/CK). The statistical analysis was performed according to Poisson distribution. The *p*-value was calculated by the following formula.

### Real-time quantitative PCR of mature miRNAs

RT-qPCR was used to validate the results obtained from the high-throughput sequencing of miRNAs. RNA that was isolated using the Trizol (Invitrogen) as described above was reversely transcribed using One Step PrimeScript miRNA cDNA Synthesis Kit (TaKaRa). This kit adds ploy (A) to the 3' end of miRNAs and start to reversely transcribe. The reverse transcription was led by a kind of special oligo-dT ligated with known sequence at its 5' end. RT-qPCR was performed using SYBR *Premix Ex Tag *II (TaKaRa) and all the primers used were listed in Additional file 5. Small nuclear RNA U6 was used as internal control. Subsequently, RT-qPCR was performed using Stratagene M × 3000P instrument.

## Authors' contributions

TW LC WZ designed the experiments; TW LC conducted the experiments; TW LC MZ QT analyzed the data; TW WZ wrote the paper. All authors read and approved the final manuscript

## Supplementary Material

Additional file 1 **The miRNA abundance of non-conserved miRNA families in control (CK) and drought stress (DS) libraries**. Click here for file

Additional file 2 Potential miRNA candidates without miRNA*s in *M. truncatula*. Click here for file

Additional file 3 **The predicted hairpin structures of all the 29 new miRNAs/new members of known miRNA families' precursors**. Click here for file

Additional file 4 **Known miRNAs in response to drought stress**. Click here for file

Additional file 5 **The primers designed for RT-qPCR**. Click here for file
