# Supplementary material for: Identification of drought-responsive microRNAs in Medicago truncatula by genome-wide high-throughput sequencing
Source: BMC Genomics. 2011 Jul 15;12:367. doi: 10.1186/1471-2164-12-367 (PMC3160423; doi:10.1186/1471-2164-12-367)
Supplement: Additional file 3 — The predicted hairpin structures of all the 29 new miRNAs/new members of known miRNA families' precursors. [file 1471-2164-12-367-S3.PDF]

Additional file 3 - The predicted hairpin structures of all the 29 new miRNAs/ new members of known miRNA families' precursors. The mature miRNA and miRNA* sequences are colored in red and blue, respectively.

miR5213

```
          10         20        30        40        50        60
UCUUA--|     U    UU-   A         U   C      CUAU   UAUU   UU  A    U
       GGCUAG GAAA   GAU CGUGUGUCU CAC UCUGAA    CAA    AUG  UC AAUC \
       UCGAUC CUUU   CUA GCACAUAGA GUG AGACUU    GUU    UAC  AG UUAG U
UUAUCCA^     -    UCU   C         C   -      C---   UUCU   UU  C    U
         120        110       100         90           80        70
```

miR5274b

```
         10        20        30        40
-|     CG                         A      GA  UA
 GCGUUC  CAAUAUGACGGAGUGUAAAUGCCUA GUAUCA  CU  C
 CGCAAG  GUUAUACUGCCUCACAUUUACGGAU CAUAGU  GA  C
A^     AU                         A      UC  UA
    90        80        70        60        50
```

miR5554a

```
         10        20        30        40         50
-      G     U      UG   A             G      -|   CA
 CAGCAU CGAUG GCAUCU  AAC AUGGUAUUCAAGU UCAAGA AAUG  \
 GUUGUA GCUAC CGUAGA  UUG UACCAUAAGUUCA AGUUUU UUAC  C
G      G     U      CG   C             A      C^   UC
   100        90        80        70        60
```

miR5554b

```
         10        20        30        40         50
CA-    G     U      UG   A                -|       CA
   GCAU CGAUG GCAUCU  AAC AUGGUAUUCAAGUGUC AAGGAAUG  \
   UGUA GCUAC CGUAGA  UUG UACCAUAAGUUCACAG UUUCUUAC  C
GGC    G     U      CG   C                U^       UC
   100        90        80        70        60
```

miR5554c

```
         10        20        30        40         50
CA-    G     U      UG   A                -|       CA
   ACAU UGAUG GCAUCU  AAC AUGGUAUUCAAGUGUC AAGGAAUG  \
   UGUA GCUAC CGUAGA  UUG UACCAUAAGUUCACAG UUUCUUAC  C
GGC    A     U      CG   C                U^       UC
   100        90        80        70        60
```

miR5555

```
        10        20        30           40
UAC              A                   ---   -|  UU
   AUGUAUCUAAGAGU UAAUAUGACUUUGAUUAUG   ACA CAC  \
   UACAUAGAUUCUCA AUUAUGCUGAAGCUAAUAC   UGU GUG  U
AAC              C                   CGU   A^  AG
    90        80        70        60        50
```

miR5556

```
         10          20          30          40          50
GU                A          C     C   C         A    AA    -----|   C   A
  CAAGAUCAUUUGGA UUCUUCCG CAUC AA GAACCUU GAG  CAG      CUC AC A
  GUUCUAGUAAACCU AAGAAGGC GUAG UU CUUGGAA CUC  GUU      GAG UG U
UU                A          A     U   A         C    CA    CCUCA^   U   A
          110         100         90          80          70          60
```

miR5557

```
        10         20         30            40
C      C  C                            .-A|     C
 AGGACA UU UCAACAAGUACUAAGGAAGCACAAUCAG   AAGUAA U
 UUCUGU AA AGUUGUUCAUGAUUCCUUCGUGUUGGUU   UUCAUU C
-      C  U                            \ -^     U
    310        300        290        280           50
```

```
                          60          70          80         90         100
--------------------       CC  .-UU     CA--      CAU   CCU   .-CUUC      UCA
                    UGUUGUG  CA     CAGUG    UUUAGC    CAA   CUC      UGAACA   \
                    AUAAUAC  GU     GUCAC    AAGUUG    GUU   GAG      ACUUGU   C
GAAGAAAUAAGCUUUUGACU       UU  \ --     UUAG      AU-   UAC   \ ----      UUA
     270        260        250               200        190                110
```

```
120         130         140         150
CUACU   UU-      CA         GUUUC      AUC
     GAU   CAAAU   UCUCUUU       ACAAG    A
     CUA   GUUUG   AGAGAAA       UGUUU    U
U----   UGU      --         -----      AAC
     180            170              160
```

```
210        220
  UCAA       UU       CU
      UUUGGG   AGUGUA   \
      GAAUCC   UCACGU   C
  UC--       --       UU
         240
```

miR5558

```
        10        20        30        40
U|    CAUA     C               CCUAU      C
 GACCU    UUUUC AAUUCUAAGUCUAUC     GAAUUC A
 CUGGA    AAAAG UUAAGAUUUAGAUAG     CUUAAG A
C^    AUCG     A               AGC--      U
     80        70        60           50
```

miR5559

```
        10        20        30            40
UAUU                  U        A      .-A|    CAA
    UCCUUUUACUUGGUGAAU GUGGAUC UUCUGU   UCAAU   \
    AGGAAAAUGAACCACUUA UAAUCUAG AAGACA  AGUUA   C
GCAU                  U        C      \ -^    CAC
      130       120       110                 50
                                            60
                                        AG--    .-A   AU
                                            CUUC   UGU  \
                                            GAAG   ACA  A
                                        CAAA    \ -   GA
                                      100             70
                                                        80
                                                  A--  UCAA
                                                     GA     C
                                                     CU     G
                                                  AAA  UAUA
                                                   90
```

miR5560

```
        10         20        30         40        50
A     C   C    -      CUC              -|   C      A   G
 GUCGG AAU CUCU CAUUCA   AGCCGGUACAUCAU UGGU GUUGGA UUU A
 CAGUC UUA GAGG GUAAGU   UCGGCCGUGUAGUA AUCA CAACCU GGA U
-     U   A    C      AAC              C^   A      A   C
110       100        90        80        70        60
```

miR5561

```
         10        20        30        40
-|   A                 C       AA      U    G
 UGGA ACUUUCAUUUGGAGAGA AUAGACA  UGAAAU AAAU A
 ACCU UGAAGGUAAAUCUCUCU UAUCUGU  ACUUUA UUUA U
U^   G                 C       A-      -    A
        80        70        60          50
```

miR5562

```
        10        20        30          40          50
AUAAAACA     GG        G    A       --     .-AAUGGGA|    G
        GUUGU  AGUCUUUU CAUG AGUUUCU  UGCUG         CGGUG C
        CAACG  UCGGAAGA GUGU UCAAAGA  ACGAU         GCCAU A
UCAUACUC     --        G    A       AA     \ -------^    A
  220         210       200       190                60
```

```
       70        80            90       100
AAAUG-        CAAAG    .-G    .-AAACAACGAGAAA    GGG
      GAAUCGUU     AUUG   UCCA               GCAU   \
      CUUAGCGA     UAAC   AGGU               UGUA   A
AACGAA        AUUA-    \ -    \ -------------    AGA
 180       170
```

```
120
GAG    GC
   GGUU  \
   UCAA  A
A--    AA
    130
```

```
140       150
 UUUGA    UGG
      GAAU   A
      CUUG   A
 AA---    UUU
160
```

miR5563

```
         10        20        30        40
-     UUA        C                      AC---|    CA
 CAAUU   AAUGAUAU AGGCAACUCGGUCCUUCUGUUA     UUGGC  \
 GUUAA   UUGCUGUA UCCGUUGAGUCAGGAAGGCAAU     AACUG  A
G     UAC        A                      GACCA^    UC
          90        80        70        60        50
```

miR 2592a

```
        10        20          30        40        50        60        70        80        90
G   C         C           -|             GAA                  G  C       AAU      C  U  C   U   A  AG
 UCA GCAUUUCGC CGGCAUUCAUG UUUUCCUUUGAAAA   UAAAUUUUUGUUAGGU UG UUUAGAU   GGUAUU AA UG CAA GAA UG  \
 AGU CGUAAAGCG GCUGUAAGUAC AAAAGGAAACUUUU   AUUUAAGAACAAUCCA AC AAAUCUA   CCAUAA UU AC GUU CUU AC  G
-   U         A           A^             AUG                  -  C       CUU      -  C  A   C   -  UU
   190       180       170       160       150       140        130       120        110        100
```

miR 2592bl

```
        10        20         30        40        50        60
AG|                      U  CU      A   UAA          U          G
  UUGUUGUUUGGCAAGUUUGAAUU AC  CAUUCA AGG   GAUAAUUGUU UAGUUGGAAG U
  GACGGCAAAUUGUUCAAACUUAA UG  GUAGGU UCC   CUGUUAACAA AUCAACCUUC G
AG^                      -  AG      C   UC-          C          C
        120       110        100        90         80        70
```

miR2592bm

```
        10        20        30        40        50        60         70         80        90
C                         U                U      C      G   GA-         AU   -|           UG      A
 UCAAGCAUUUCGCUCGGCAUUCAUG UUUUCCUUUGAAAAGA UAAAUU UUGUUA GUU   UUUAGAUGA  GUA UUAAGUGUCAA  AAUGA \
 AGUUCGUAAAGUGGGCUGUAAGUAC AAAAGGAAACUUUUCU AUUUAA AACGAU CGA   AAAUCUACU  UAU AGUUCACGGUU  UUAUU C
-                         -                U      A      G   ACA         AC   A^           GU      C
  190       180       170        160       150       140       130       120       110       100
```

miR2592bn

```
         10         20         30         40         50         60          70          80          90
C                          U               UAU       C          -  G          A    -           -|      AA
 UCGAGCAUUUCGCUCGGCAUUCAUG UUUUCCUUUGAAAA   UAAAUU UUGUUGGGU UG UUUAGAUGA GGUA UUAAGUGUCAA GAAAUG   \
 AGUUCGUAAAGUGGGCUGUAAGUAC AAAAGGAAACUUUU   AUUUAA AACAAUCCA AC AAAUCUACU CCAU AGUUCACGGUU CUUUAC   C
-                          -               CUU       A          C  G          A    A           G^      UC
   190        180        170         160        150        140        130        120        110        100
```

miR2619b

```
         10         20         30         40           50         60         70         80
AAA|       A     U                      GC       .-AGGCAAAAUAAAAAUAAAAAAUUUGACACA        CA
   CAGCCCC UAUGU UUGAUUCUUUGGCAGUUUUG  CCCCCA                                  UGGCACCU  C
   GUCGGGG AUACA AACUAAGAAACCGUCAAAAC  GGGGGU                                  ACCGUGGG  U
UUA^       G     U                      UU       \ ------------------------------        AU
          160        150        140        130                                        90
                                                  100        110
                                                  CG     C       C
                                                    UCAG GUUGAC \
                                                    AGUC CAACUG G
                                                  CA     A       A
                                                      120
```

miR156j

```
           10          20         30         40         50         60
--|    G UG  A   A-        A        GG         A     GUCUUUA     UAA
  CUGUU U  GG CAU  GAAAUUG CAGAAGAG  UGAGCACA AAAAA       GUAUA    \
  GAUAA A  UC GUA  UUUUAAC GUCUUCUC  ACUCGUGU UUUUU       CAUAU    U
GU^    - GU  -   AC        A        AU         G     AUUAC--     UUG
    120          110        100         90         80          70
```

miR167b

```
        10        20        30        40        50        60        70        80        90       100       110
CAA|     U       G              G   U  UUCCUGUGU   U    UAAU      CUGA            C      A   A   U        UAA
   UGACAG UGAAGCU CCAGCAUGAUCUGU CUU CC         AUA ACUU    UUCCAG    AUUUAAAUAUAA CAAAAA AUA AUA GUUUGGUC   A
   AUUGUC ACUUCGA GGUUGUACUAGACA GAA GG         UAU UGAG    GGGGUC    UGAAUUUGUAUU GUUUUU UAU UAU CAAACUAG   U
UAA^     C       -              A   U  UGUU-----   U    U---      ----            C      A   A   U        UUU
     200        190       180       170            160              150       140       130       120
```

miR168c

```
            10        20        30        40        50        60
C----|   UU         UA     C          U     A   CUA  U      U    G
     UCAC  CGCGGUCUC  AUUCG UUGGUGCAGG CGGGA CCA   CA CCGCUG UUUC \
     AGUG  GCGCCGGAG  UAAGU AACUACGUUC GCCCU GGU   GU GGCGGC AAAG U
AUCUA^   --         GC     C          C     A   UAA  -      C    A
    120         110       100        90          80        70
```

miR172b

```
          10        20        30          40        50
CAC--      UGUUU  A                     .-AUAUGUGAAUGAU|   AGU
     AGUCGU     GC GAUGUAGCAUCAUCAAGAUUC               GCAG   \
     UCAGCA     CG CUACGUCGUAGUAGUUCUAAG               CGUC   G
AUAUU      UAUAC  A                     \ -------------^   AAG
  130       120       110       100                         60
```

miR172c

```
        10        20        30         40        50        60        70
UUAUUU  A                     A  UA-|     U  AA     UUUUG        UG   UG
      GC GAUGUAGCAUCAUCAAGAUUC CA   UGAAAG GC  AUGG     UUGGAUUU  AUC  \
      CG CUACGUCGUAGUAGUUCUAAG GU   GUUUUC UG  UACC     AAUCUAAA  UAG  A
AAAUAC  A                     A  CAA^     -  GA    UCUUG        CG   CU
         130       120       110       100         90        80
                                            70        80
                                          UAA        UUG
                                             UAUUUCAA   A
                                             AUAAAGUU   U
                                          A--        UUC
                                               90
```

miR408

```
        10         20        30        40        50        60
UAGU   U      ACA-|  A       CAU    A     GA  AAA   U       GAA
    UAA GACAGG    AAG CAGGGAA   GCAG GCAUG  UG   CUA CGACAGU   \
    AUU UUGUCU    UUC GUCCCUU   CGUC CGUAC  AC   GGU GUUGUCA   G
UAUU   U      AAUA^  G       CUC    A     UC  CUC   -       AAG
   120       110       100        90        80         70
```

miR2111u

```
         10        20        30         40         50
-     U             UC UG        -      AGU-| GU  A    CUU
 AAGGA AGGGUAAUCUGCA  C  AGGUUUAG AACAAU    UG  UA UUUC   A
 UUCCU UUCUAUUAGACGU  G  UCCGAAUC UUGUUA    GC  AU AAAG   A
A     U             GA GU        U      AUGC^ UG  A    AAA
   110       100        90        80        70        60
```

miR2111v

```
         10         20        30        40         50
UA      -   C  AU-              C UG         -|  ACC  U
  UAUUGG GAU AG   GAGAUAAUCUGCAU C  AGGUUUAGA ACA   GA G
  GUAACC CUA UC   CUCUAUUAGACGUA G  UCCGAAUCU UGU   CU C
AC      A   U  CUU              A GU         G^  CA-  G
      100        90        80        70        60
```

miR2643b

```
           10        20        30        40        50
CUC---|  U   CC    AU        C    U      UUA     A   CAU
      UGC AGU  UUGA  UCUCUAAU UCUG UCCCAA   CACUU AUC   G
      ACG UCA  AACU  AGAGAUUA AGAC AGGGUU   GUGAA UAG   A
AGAUCA^  U   U-    AU        A    U      UGA     C   AAA
        100         90        80        70        60
```

miR4414a

```
          10        20        30        40        50
GUAGAA--        AG CU     G   AC       U   AU   U    -----|   GC
        GAUGGCUG  A  CAGCU CUG  UCGUUGG UCA  AGC CACA     ACAU  \
        CUGCUGAC  U  GUCGA GGC  AGCAACC AGU  UCG GUGU     UGUG  A
UACUGUCA        CU AC     G   GU       U   CU   U    GCAAC^   AU
120       110       100        90        80        70        60
```
